# An Interdisciplinary Approach for Management of an Extensive Carious Premolar

**DOI:** 10.22037/iej.v13i3.20871

**Published:** 2018

**Authors:** Wong Lishen, Tew In Meei, Alizae Marny Mohamed, Dalia Abdullah

**Affiliations:** a *Centre for Restorative Dentistry, Faculty of Dentistry, The National University of Malaysia, Jalan Raja Muda Abdul Aziz, 50300 Kuala Lumpur, Malaysia;*; b * Centre for Family Oral Health, Faculty of Dentistry, The National University of Malaysia, Jalan Raja Muda Abdul Aziz, 50300 Kuala Lumpur, Malaysia*

**Keywords:** Interdisciplinary Treatment, Orthodontic Extrusion, Root Canal Treatment

## Abstract

The principle of ferrule effect is of prime importance when restoring an endodontically treated tooth. A severely broken down tooth due to subgingival caries almost always end up with extraction as inadequate ferrule effect would compromise the predictability of restorative treatment. This clinical case report describes a treatment approach that combines non-surgical endodontic treatment, orthodontic extrusion and prosthetic rehabilitation to restore the function and aesthetic aspect of an extensively carious premolar with compromised prognosis. One year follow-up indicated stable periodontal health with evidence of periapical healing radiographically.

## Introduction

Restoring a non-vital tooth with subgingival caries is always a challenge. Such a tooth would eventually be extracted due to poor long-term prognosis. In order to ensure optimal restorative outcome, provision of adequate coronal tooth structure at crown-root interface is of great importance. Although the concept of partial ferrule is well described [1], a uniform and full “all around” ferrule has, in a significant manner, shown superiority in fracture resistance of an endodontically treated tooth [2].

Surgical crown lengthening is usually performed to facilitate restoration of tooth with subgingival lesions. However, this surgical approach may lead to reduced crown-root ratio and bony support due to bone removal [3]. In addition, loss of interdental papillae and presence of “black triangle” consequential of crown lengthening may yield un-aesthetic result [4].

Therefore, orthodontic extrusion of tooth may be valuable in certain clinical situation as an alternative approach. 

The aim of orthodontic extrusion would be to relocate the restoration margin to the level above the gingiva, thus enabling provision of an extracoronal restoration that would not encroach biological width. The traction forces that are applied on the periodontal ligament would also help to stimulate marginal apposition of crestal bone as well as coronal shift of marginal gingiva. Hence, the aesthetic outcome of the final restoration could be assured. 

The purpose of this article is to describe management of a maxillary left first premolar with advanced subgingival caries by means of an interdisciplinary approach including an endodontic, orthodontic and prosthodontic team.

## Case Report

A 42-year-old male patient was referred to the Endodontic Specialist Clinic in 2016 for the management of left maxillary first premolar with advanced caries. At the time of consultation, tooth #24 was asymptomatic. The patient’s medical history was non-contributory. 

**Figure 1:**
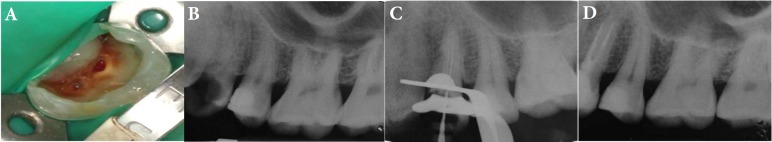
*A*) Occlusal view of tooth 24; *B*) Preoperative radiograph; *C*) Working length radiograph; *D*) Postoperative of tooth 24 with fiber post and composite core placement

**BAFigure 2:**
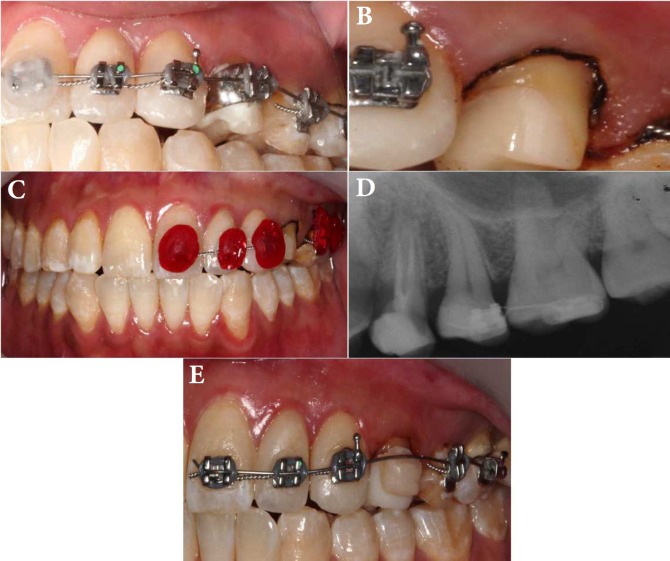
*A*) Buccal view of orthodontic apparatus; *B*) Post-retained all ceramic crown preparation; *C*) Beading wax was placed on the orthodontic brackets to ease final impression for the crown; *D*) Periapical radiograph eight weeks after orthodontic extrusion; *E*) Composite button was placed on provisional crown

**Figure 3:**
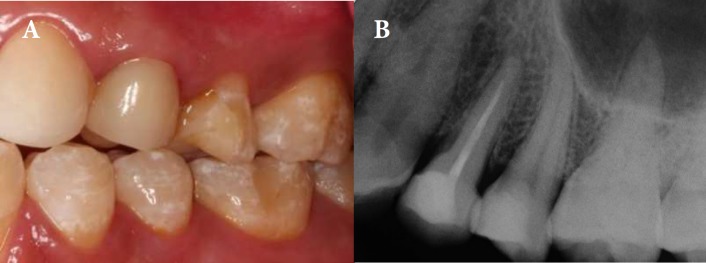
*A*) Buccal view of tooth 24; *B*) Periapical radiograph one year after treatment completion

Clinical examination revealed subgingival caries on mesial surface of tooth #24 (Figure 1A). The probing depth was within normal limit. The tooth was not tender to percussion, palpation or biting and it was not mobile. Pulp sensibility test (Elements Diagnostic Unit, SybronEndo, Orange, CA, USA) was performed and failed to elicit a response. A periapical radiographic examination revealed a periapical radiolucency of the tooth (Figure 1B). The tooth was diagnosed with pulp necrosis; asymptomatic apical periodontitis. 

The patient was advised of the clinical findings and various treatment options were discussed. The patient decided to proceed to the following agreed treatment plan which was; 1) nonsurgical root canal treatment, 2) orthodontic extrusion by a sectional fixed appliance and 3) post-retained all ceramic crown. The potential technical difficulties such as providing coronal seal and exposure of the sound tooth structure *via *orthodontic extrusion for placement of restorative margin were explained to the patient.

Written consent for the proposed treatment was obtained from patient. At the first visit, nonsurgical root canal treatment was initiated after local administration of 2% mepivacaine with 1:100000 epinephrine (Scandonest 2% Special, Septodont, France). The tooth was isolated with dental dam. The canals were accessed under surgical microscope (OPMI Pico Zeiss Dental Microscope, Germany). The working length was determined using an electronic apex locator (Root ZX mini, J. Morita, Japan) and verified radiographically (Figure 1C). The canals were prepared using NiTi rotary files (ProTaper NEXT rotary files, Dentsply Maillefer, Ballaigues, Switzerland). Sodium hypochlorite (2.5%) was used as an irrigant and calcium hydroxide (Calcipex II, Nishika, Japan) as intracanal medicament. The access cavity was restored with temporary restoration (Intermediate Restorative Material, Dentsply Caulk, Milford, United States). After a week, the root canals were re-entered and irrigated with 2.5% sodium hypochlorite to remove the intracanal medicament. Fitting of master gutta-percha was verified radiographically. Canals were irrigated with 2.5% sodium hypochlorite, 17% ethylene-diamine-tetra-acetic acid, normal saline and 2% chlorhexidine (final irrigation protocol) using passive sonic irrigation (EndoActivator System Kit, Dentsply Maillefer, Ballaigues, Switzerland). The root canals were dried with calibrated absorbent paper points. Warm vertical compaction technique was used to obturate all canals. After the completion of obturation, post space was prepared in the palatal canal using post drill. Five mm of gutta-percha was retained apically and fiber post (RelyX™ Fiber Post, 3M ESPE, United States) was cemented with resin cement (RelyX™ Unicem™ Self-Adhesive Universal Resin Cement, 3M ESPE, United States). Subsequently, composite core was placed on the tooth (Figure 1D). 

The patient was then scheduled for an orthodontic extrusion procedure by using a segmental arch techniques (SAT) of fixed appliance. An orthodontic band with labial bracket was fitted and cemented on tooth #24 to allow all remaining crown structures to be protected for retention and resistance during extrusion. Brackets were bonded on the labial surfaces of teeth #21 to 26 (Figure 2A). These neighbouring teeth were used as anchoring teeth. An orthodontic wire (0.016 NiTi) was ligated to the bracket from tooth #21 to tooth #26 to produce the extrusion force on tooth #24, so that the NiTi wire was able to provide a continuous low force for a long time frame. After four weeks, tooth #24 had been extruded for about 1 mm. The same band was debanded and recemented more gingivally. The extrusion force was reapplied with the same arch wire and palatal cusp tip of tooth #24 was reduced to prevent occlusal interference with the lower teeth. After the second four weeks, around 2 mm of extrusion had been achieved and this was judged to be sufficient (Figure 2B). 

Following extrusion, fibrotomy was performed under local anaesthesia to prevent relapse. Subsequently, post-retained all ceramic crown preparation was performed and acrylic crown was cemented as provisional crown (Figures 2C and 2D). Composite button was placed on provisional crown to retain the position of the extruded tooth #24 (Figure 2E). All ceramic crown was cemented as the final restoration a week later. 

At one year review, patient was symptom-free. He expressed his satisfaction with the treatment from aesthetic point of view (Figure 3A). Clinically, the crown was satisfactory and there was no evidence of apical pathology in periapical radiograph (Figure 3B). 

## Discussion

The present case report describes the successful outcome of an interdisciplinary approach, including non-surgical endodontic treatment, orthodontic extrusion and prosthodontic rehabilitation in management of an extensive carious maxillary premolar.

The remaining tooth structure is the most important predictor for long term success of restoration on endodontically treated tooth. Limited tooth structure in this case has posed challenges to prosthetic rehabilitation. The subgingival margin placement at the caries site increases gingival inflammation and loss of clinical attachment [5]. 

In order to ensure functional longevity, relocation of restoration margin to a level above gingival margin is necessary, not only to improve the ferrule effect but also to promote a more favourable gingival and periodontal response [6, 7]. Surgical crown lengthening or orthodontic extrusion have been well documented to serve this purpose [7, 8]. Orthodontic extrusion was recommended in this case as it could maintain the crown-root ratio of the tooth and provide good gingival and osseous profiles which are critical in aesthetic zone [8]. If surgical crown lengthening is performed on a single tooth to relocate the margin such as in this case, the main problems to be addressed are the extent to which the periodontal support has to be removed both in relation to the tooth in question and to the adjacent teeth, and the imbalance between gingival levels that could occur at the completion of the treatment which would affect the aesthetics.

A few studies have investigated on the acceptable ferrule height for an endodontically treated tooth [1], Pereira *et al*. [10] found that the higher the ferrule height, the greater the fracture resistance of the tooth. However, minimum of 1 mm ferrule height is suggested to be able to increase the fracture resistance of endodontically treated tooth by two folds [9]. Thus, extrusion of 2 mm in this case has successfully provided two mm ferrule height which is considered adequate to promote good periodontal health and facilitate fabrication of a post-retained crown. 

Fiber post plays an important role to achieve an aesthetically favorable restorative outcome. The use of fiber post will reduce the risk of root fracture as its inherent modulus of elasticity closely matches that of the dentine [11]. Although, the duration of fiber post cementation after root canal treatment remained controversial, it was placed immediately after endodontic treatment in this case as delayed post space preparation resulted in more apical leakage than immediate preparation [12]. Apart from that, it is also worth taking note that the flexibility of fiber post may, however, lead to marginal leakage [13] or fracture of the post but the risk is minimized in this case with provision of adequate ferrule height [10].

The use of orthodontic extrusion has been suggested as an alternative conservative approach for periodontal crown lengthening that allows placement of a crown on a sound tooth structure. There is no uniformity in the literature on various strategies used for orthodontics extrusion as it depends on clinical situations [14, 15]. Basically, it is necessary to provide a stable anchorage that acts as a support for the transmission of the forces on the tooth/teeth to be extruded. In this case, the SAT consisted of a combination of teeth in an active unit and a passive unit (anchor). The anchor block provided maximum stability of anterior and posterior teeth to tooth #24, during extrusion movement. This enabled effective reciprocal forces and moments acting on tooth #24 to be controlled and extrusion to be achieved. In addition to this technique that can reduce unwanted effects without patient co-operation, it is easily carried in the posterior area without the aesthetic sequelae. Following orthodontic extrusion, the marginal periodontal fibres are stretched (tension). This causes reversal of the osseous architecture and the relapse of the extruded tooth. However, the risk in this case is minimized through immediate fibrotomy (incision of suprecrestal fibres). Incision of the free and transeptal fibres which was performed in this case reduces this risk even further [16]. 

## Conclusion

This case report was intended to share information on interdisciplinary approach to manage an advanced subgingival carious tooth with compromised prognosis. Orthodontic extrusion has been very beneficial in the establishment of biological width, maintaining the periodontal support of the adjacent teeth and restore aesthetics. Therefore, orthodontic extrusion could be a useful treatment option to consider, to retain the tooth as long as possible. 
